# Record of Meteorology and Epidemics of Buffalo and Vicinity, for July, 1854

**Published:** 1854-09

**Authors:** Sanford B. Hunt


### ART. II.—*Record of Meteorology and Epidemics of Buffalo and Vicinity, for July*, 1854. By Sanford B. Hunt, M. D.

| Date. | Inches on Barometer. | Inches of Rain. | Amount and | Course of Clouds. | Force and Course | of Wind. | Upper Station. Hygrometer. Temp. | Upper Station. Hygrometer. Dew-point. | Upper Station. Hygrometer. Humidity. | Lower Station. Hygrometer. Temp. | Lower Station. Hygrometer. Dew-point. | Lower Station. Hygrometer. Humidity. | Deaths from Cholera. In City Proper. | Deaths from Cholera. At Poor House. |
|---|---|---|---|---|---|---|---|---|---|---|---|---|---|---|
| 1 | 28.9 | | 0 | | 1 | S. W. | 77 | 60.66 | 616 | 74 | 62.33 | 693 | 3 | |
| 2 | | | 0 | | 1 | S. W. | 78 | 59.33 | 565 | 80 | 73 | 811 | 3 | |
| 3 | 29.3 | | 1 | N. N. E. | 0 | S. S. W. | 79 | 67.33 | 686 | 78 | 68.66 | 761 | 3 | 1 |
| 4 | | 0.65 | 2 | | 1 | S. W. | 80 | 57 | 517 | 81 | 67 | 657 | 3 | |
| 5 | 29.2 | | 0 | | 1 | S. W. | 79 | 67.33 | 686 | 74 | 62.33 | 693 | 2 | 1 |
| 6 | 29.2 | | 0 | | 1 | S. W. | 79 | 60.33 | 564 | 77 | 63 | 654 | 2 | |
| 7 | 29.2 | | 0 | | 1 | S. W. | 83 | 62 | 532 | 80 | 64 | 618 | 1 | |
| 8 | 29.2 | | 0 | | 1 | S. W. | 83 | 71.33 | 702 | 82 | 62.66 | 766 | 4 | |
| 9 | | | 5 | E. N. E. | 2 | W. S. W. | 77 | 60.66 | 616 | 72 | 58 | 657 | 2 | |
| 10 | 29.2 | | 1 | E. | 2 | W. S. W. | 75 | 56.33 | 563 | 74 | 53 | 530 | 1 | 1 |
| 11 | 29.2 | | 0 | | 2 | W. | 77 | 59.66 | 616 | 75 | 62.33 | 694 | 3 | 2 |
| 12 | 29.2 | | 1 | S. E. | 3 | W. S. W. | 72 | 53.33 | 560 | 76 | 57.33 | 563 | 3 | 1 |
| 13 | 30 | | 1 | E. | 2 | N. W. | 73 | 54.33 | 558 | 74 | 53 | 530 | 5 | 1 |
| 14 | 29.2 | | 2 | N. W. | 1 | W. | 77 | 56 | 530 | 76 | 59.33 | 614 | 4 | 2 |
| 15 | 29.2 | | 1 | | 2 | S. W. | 80 | 63.66 | 618 | 79 | 62.33 | 607 | 6 | 2 |
| 16 | | | 3 | N. E. | 1 | S. W. | 82 | 65.66 | 620 | 84 | 65.33 | 620 | 3 | 1 |
| 17 | 29.2 | | 3 | | 2 | W. S. W. | 85 | 64 | 528 | 84 | 63 | 503 | 4 | |
| 18 | 29.2 | | 3 | | 1 | S. W. | 82 | 65.33 | 620 | 80 | 68.33 | 697 | 1 | 2 |
| 19 | 29.2 | | 5 | S. E. | 1 | S. W. | 84 | 72.33 | 702 | 83 | 71.33 | 702 | 2 | 9 |
| 20 | 29.1 | 1.06 | 3 | E. | 2 | W. S. W. | 84 | 70 | 660 | 84 | 72.33 | 702 | 5 | 2 |
| 21 | 29.2 | | 4 | | 1 | W. | 86 | 72 | 663 | 86 | 69.33 | 620 | 8 | 2 |
| 22 | 29.1 | | 2 | | 2 | N. E. | 84 | 72.33 | 702 | 82 | 70.33 | 691 | 5 | 2 |
| 23 | | 0.28 | 0 | | 1 | N. E. | 81 | 69.33 | 697 | 78 | 68.66 | 761 | 11 | 1 |
| 24 | 29.2 | | 7 | E. | 1 | W. | 82 | 61 | 533 | 85 | 64 | 528 | 8 | 7 |
| 25 | 29.2 | 0.70 | 0 | | 1 | E. S. E. | 85 | 71 | 661 | 84 | 63 | 532 | 14 | 3 |
| 26 | 29.1 | | 0 | | 1 | E. | 77 | 56 | 530 | 77 | 58.33 | 565 | 10 | 3 |
| 27 | 29 | | 0 | | 4 | S. W. | 76 | 57.33 | 565 | 73 | 60 | 695 | 15 | 9 |
| 28 | 29 | | 0 | | 1 | S. W. | 77 | 56 | 530 | 74 | 60 | 657 | 7 | |
| 29 | 29 | 1.45 | 3 | N. N. E. | 2 | S. W. | 78 | 60.33 | 686 | 78 | 68.66 | 761 | 10 | 1 |
| 30 | | | 1 | | 3 | S. W. | 78 | 59.33 | 565 | 79 | 62.33 | 609 | 7 | 1 |
| 31 | 30 | 1.37 | 1 | | 1 | S. W. | 79 | 65 | 657 | 78 | 64.33 | 655 | 6 | 1 |
| | 29.2 | 5.51* | | | | | 79.67 | 62.78 | 509 | 78.80 | 64.00 | 650 | 159 | 55 |

* Total.

The foregoing table gives an abstract of the meteorological conditions of the month of July for each day. The following explanations are needed:

1st. *The Barometer.* The height is given in inches, and tenths of inches.

2d. *Rain.* The quantity falling is expressed in inches and hundredths of inches.

3d. *Clouds.* The first column indicates the amount of clouds; as 1, covering one-tenth of the sky; 3, covering three-tenths; and so on. The direction towards which the clouds moved is given in the usual letters, marking points of the compass.

4th. *Winds.* Their force is indicated by the first column; 1 stands for a very light breeze; 2 for a gentle breeze; 3 for a fresh breeze; 4 for a strong wind; indicating a velocity of 2, 4, 2, and 25 miles per hour, respectively. The point of compass is expressed in the usual manner.

5th. The Hygrometer needs no explanation.

6th. The numbers of deaths given are from official reports, and are accurate.

*Remarks.* The meteorology of July 1854 derives especial interest from the fact that this month has witnessed the development of an epidemic of remarkable extent, and, in many places, of unusual fatality. Within our personal observation, three distinct epidemics have occurred, in such varying locality and circumstances as afford some data for comparison, especially as the hygrometic condition of the atmosphere must have been nearly the same in each locality.

The indications afforded by the barometer are not important. The specific gravity of the atmosphere varies from so many causes that it is a very uncertain guide. Its range of variation during the month was very slight, and had no apparent connection with the fall of rain or wind.

*Rains.* The quantity falling during the month was large, the sum being 5, 51 inches. The dew point was usually high on the days when rain fell, and declined upon the succeeding day slightly.

The observations on the amount of clouds show that the sky was generally clear. None of the rains were of long duration.

*Winds.* The wind was south-west on sixteen days, south-south-west on one day, west-south-west on five days, west on four days, north west on one day, north-east on two days, and easterly on two days.

The highest humidity accompanied south-west winds, though the north-east wind on two days was attended by great heat and humidity.

*Temperature.* The highest heat was 86°, the lowest 72°, the average 79°, 67. The average dew-point was 62°, 78. Its highest range was 72°, 33, its lowest 56°. There were six days on which the dew-point was above 70°; which is but a little below the usual New Orleans average for the same month.

The differences between the upper and lower stations are noteworthy. The temperature is 0°, 87 higher at the upper station, the dew point 1°, 22 lower, and the humidity is lower by the fraction .041. These differences depend upon a difference in altitude and locality of 45 feet.

In tracing the connection of humidity with cholera we shall use the dew-point as the essential condition. The dew-point being high, the humidity is almost necessarily so; while the relative humidity may be large, with but a small amount of moisture, inasmuch as the relative humidity is only the answer to this proposition:

As the amount of vapor which the air can contain at a given temperature is to the amount it does contain, so is 1000 to the relative humidity.

We find in the opening days of the month a few cases of cholera occurring. Up to the 19th not over six deaths occurred upon any day, and the average was considerably less than that. The dew-point during this time was below 60°, seven days; below 65°, and over 60°, six days; below 67° and over 65°, four days. On the 8th it reached 71° for a few hours only.

On the 18th, cholera appeared at the county poorhouse, a dew-point of about 65° having obtained for four days previous. On the 19th with the dew-point at 72°, nine* deaths occurred. The mortality continued during the month until *one-fifth* (55) of the population (about 279) of this lazar-house had perished. Up to the 25th the dew-point ranged at 70° and above that figure, with one exception. A corresponding mortality occurred on some of the unpaved and undrained streets in the poorer parts of the city. On Mortimer street, containing 25 houses, 17 tenements had 50 cases of cholera and 20 deaths.

On the 26th the dew-point suddenly fell to 56°, with a striking decrease in new cases; though, as will be seen on reference to the table, the mortality from cases originating prior to that date continued, there being 11 deaths in the city, and 3 at the poor-house, on the 26th; and 14 in the city, and 9 at the poor-house, on the 27th.

The dew point averaged below 60° for the remainder of the month, (from the 26th to the 31st) and its effects was marked by a sudden decrease in the deaths (on the 28th, 8; 29th 6; 30th, 7; 31st 4.) During these days there were only 3 deaths at the poor-house.

It is with no little interest that we turn to another locality —— Suspension Bridge. Isolated cases of cholera had occurred there for a few days prior to the 19th, when it assumed an alarming character, cotemporaneous with the mortality at the poor-house. During the high dew-point which obtained from the 19th to the 25th, the mortality was very great; as we know

*In stating the number of deaths at the poor-house we follow the official report of the attending physician. It does not coincide with the positive statements of some four or five of the employees, as made to the Board of Health. But we are not responsible for any concealment or alteration of returns which may have been necessary to patch up official character.

from personal observation on the spot; but no accurate statement of the number of deaths can be made.

On Saturday, the 22nd, the air of the place was reeking with humidity; and several showers, followed by a hot sun and a still air, must have brought it almost or quite to a dew-point of 80°. The disease continued with unabated severity until the 26th. On the morning of that day it was found that no new cases had occurred during the night on the American side. A cool easterly wind was blowing toward the Canada shore, the dew-point dropping suddenly down from 71° to 56°, while the thermometer fell only from 85° to 77°.

This change was permanent upon the American side. Even at the early hour of the 26th on which we left the scene, we were able to see a marked tendency to convalescence in those already sick, and up to July 31st this favorable condition continued. So far as our information now extends, but very few deaths occurred in that period — among them, alas! one whose devotion and fortitude will form the subject of a separate notice in this number.

We propose, at the risk of tediousness, to set side by side the meteorology and sanitary conditions which marked these three localities, viz., the city of Buffalo, the Erie County Almshouse, and Suspension Bridge.

Here are three places in the same meteorological condition, having the same winds, the same rains, and the same dew-point.

One of them is a city with a large floating and foreign population, numbering at least 80,000 souls. In all this mass of humanity, packed in crowded tenements, and shut in by brick walls, only 159 die of cholera within the month. Of these 159, only four or five are American residents, and the deaths occur away from the crowded portion of the city, and in those places where the town merges into the surrounding fields; where the streets are unpaved, and no sewers are constructed. The local causes of disease exist in a flat surface of clay soil, with no drainage, either natural or artificial. Evaporation is the only means of getting rid of surplus moisture; the habits of the population are not such as conduce to cleanliness; while the state of the streets and soil is such as to prevent the possibility of any efficient action on the part of the Board of Health.

We have, then, as the cholera condition here, a large undrained surface, occupied by a population uncleanly in habits and imprudent in diet, subjected to the influence of a high dew-point promoting evaporation, and the consequent absorption by the air of all tellurial gases; while it, also, by producing solar exhaustion predisposes the system to the inroads of disease Behind all these local, exciting, and avoidable causes of cholera, we have a mysterious cause of unknown production, but of which we really know as much as we do of the ultimate cause which determines any zymotic disease.

At the County Poor House, a first glance at the edifice would contradict our whole theory. It is a large, roomy, stone building, five miles out of the city proper, situated upon a ridge in the middle of a large field. No country farm house has a more healthy location.

But one fifth of its inmates have died of cholera in one month. This ratio of one in five, compared to the city mortality of one in five hundred and three, would go to indicate that pure country air is the exciting cause of cholera!

The first step inside the building helps to explain the enigma, while an examination of the diet of the house harmonizes the whole theory. We have already said something in a previous number of the peculiar ventilation of this edifice, of the manifold contrivances to economize air, to keep it safe within the walls, and use it over and over again; to make it nourishing by the steam of the kitchens and work-rooms, and the exhalations of damp undrained brick floors—the earth beneath them saturated with the filth of years—and then to convey it story by story to every part of the house, as fit food, good enough air, for paupers.

The diet of the house is equally characteristic. Cases of scurvy occurred from want of vegetable food, lying-in women had gruel two days, and pork and bread on the third after confinement. We do not choose to repeat what we have previously said on the subject. The facts stand proved before the community, and the whole country is familiar with the shame of Erie County, a shame which should rest alone on the single man who dictated, and the officers, household and medical, who lacked the courage and humanity to demand a reform.

No medical man should deem it honorable to retain the direction of a hospital, over the diet of which he has no control. In this case, a determined resistance on the part of the medical attendant, a threat or two of public exposure to the Superintendent in charge, would have saved fifty lives.

One more cholera condition in this home of the poor concludes the list. Behind the house is a large area, enclosed by a high board fence, for the recreation of the paupers. Here gather the inmates to get their fresh air, to sit stupidly under the direct rays of the sun, in a hot breezeless, treeless enclosure, withouta particle of shade except that found in two reeking, doorless privies in the rear. Thus solar exhaustion is added to their miseries.

As some proof that our statements are in no wise exagerated, it has been found necessary, at the demand of the Board of Health, to close entirely the house occupied by the insane, of whom one half have died, removing its inmates to temporary booths; and to make all the other reforms, the necessity of which has been indicated on the foregoing page.

At Suspension Bridge the same causes existed in a less culpable form. Perhaps a thousand foreigners had gathered there upon the public works, and erected their shanties. The epidemic commenced under the hill, where a large excavation had been made, springs of water cut across, and standing pools accumulated. The shanties of the laborers were built immediately upon the earth, and, as usual, but few of them were in any degree cleanly. The men themselves had labored in the sun, and suffered from the exposure to great heat and humidity. These conditions were then finally subjected to the influence of a high dew-point, developing exhalations, and enfeebling the system preparatory to disease.

It will be seen that we have named tellurial exhalations and idio-miasm among the epidemic causes. That they do frequently exert such an influence has long been our conviction. That they are essentially necessary to the production of zymotic disease is not so evident, but so far as we know, no great epidemic has existed without humidity as a local cause. In the instances cited by Ferguson, which have always been the stumbling block of the pure malarialists, we find that humidity, was evidently present. Even in the sandy plain of Rosendal, where there was no decomposition of organic matter, we find that on digging but a few inches, "excellent drinking water was found for the use of the troops."

This instance would go to show that the two conditions of heat and humidity are sometimes alone sufficient for the production of zymotic disease. The present epidemic of cholera in Buffalo points to the same conclusion. Thus far we have had no cholera where the drainage and ventilation was such as to carry off promptly all surface water. Many of the causes of this disease are therefore under control, and may be avoided by the means which have been heretofore found efficient.

---

